# Early lactate and its metabolism for predicting persistent renal failure in patients with acute pancreatitis: a retrospective observational study

**DOI:** 10.1186/s12876-025-04413-w

**Published:** 2025-11-17

**Authors:** Jianhua Wan, Huajing Ke, Wenhua He, Yin Zhu, Nonghua Lu, Liang Xia

**Affiliations:** Department of Gastroenterology, Jiangxi Provincial Key Laboratory of Digestive Diseases, Jiangxi Clinical Research Center for Gastroenterology, Digestive Disease Hospital, The First Affiliated Hospital, Jiangxi Medical College, Nanchang University, 17 Yongwaizheng Street, Nanchang, Jiangxi 330006 People’s Republic of China

**Keywords:** Acute pancreatitis, Persistent renal failure, Lactate, Lactate dehydrogenase, Prognosis

## Abstract

Acute pancreatitis (AP) is common and clinically complex, and persistent renal failure (PRF) is a severe complication. This study retrospectively analyzed 798 AP patients admitted within three days of onset and divided them into a PRF group (*n* = 111) and a non-PRF group (*n* = 687), comparing the characteristics of the two groups. Multivariate analysis showed that lactate (β = 0.340, *P* < 0.001, OR = 1.405) and lactate dehydrogenase (LDH, β = 0.002, *P* < 0.001, OR = 1.002) were significant risk factors for PRF. Stratified analysis indicated that patients with LDH ≥ 700 U/L and lactate ≥ 2 mmol/L had a higher incidence of PRF. The ROC curve analysis showed that the areas under the curve for lactate, LDH, and the combined lactate + LDH were 0.752, 0.828, and 0.866, respectively, with the highest diagnostic accuracy for the combined indicator. The characteristics of different risk groups showed that the incidence of PRF in the high-risk group was significantly higher than that in the low-risk group. The levels of lactate and LDH within 24 h of admission have a significant predictive effect on PRF in AP patients, which helps clinicians identify high-risk patients early and guide treatment decisions.

## Introduction

Acute pancreatitis (AP) is a common acute abdominal condition in clinical practice, with a wide range of disease severity, from mild self-limited inflammation to severe acute pancreatitis (SAP) involving multiple organ systems, posing a serious threat to patients' health [1, 2]. Among its complications, persistent renal failure (PRF) is a frequent and severe one, significantly prolonging hospital stays and greatly increasing the risk of patient mortality and long-term adverse outcomes [3, 4]. During the course of AP, local inflammatory responses can trigger a series of complex pathophysiological changes. The massive release of inflammatory factors can lead to microcirculatory disturbances, causing pancreatic tissue ischemia, and triggering anaerobic metabolic pathways within cells [5]. Systemic inflammatory response syndrome (SIRS) can further affect the blood perfusion and function of other vital organs, including the kidneys [6]. As an organ highly sensitive to changes in blood perfusion, the kidney is prone to pathological changes such as reduced glomerular filtration rate and tubular damage under low perfusion states, leading to renal impairment [7]. This renal dysfunction in AP patients may manifest as varying degrees of acute kidney injury (AKI), and if not promptly and effectively intervened, some patients may progress to PRF [8].

Currently, the prognosis assessment for AP patients in clinical practice mainly relies on traditional scoring systems, such as the Acute Physiology and Chronic Health Evaluation II (APACHE II) score, Ranson's score, and the Bedside Index for Severity in Acute Pancreatitis (BISAP) score [9–11]. Although these scoring systems can help physicians identify patients with severe conditions to some extent, they have certain limitations, such as some indicators being relatively delayed in acquisition and insufficient predictive accuracy for specific complications like PRF [12]. In addition, some biomarkers, such as C-reactive protein (CRP) and procalcitonin (PCT), have been studied in the diagnosis and prognosis assessment of AP, but the predictive value of a single indicator is still limited [13, 14]. Therefore, finding more specific and timely early predictive indicators is crucial for improving the prognosis of AP patients.

Lactic acid, being the product of anaerobic metabolism, directly reflects tissue perfusion and cellular metabolic status in the blood [15]. In the early stage of AP, due to local and systemic ischemia-hypoxia, lactate production tends to increase [16]. Lactate dehydrogenase (LDH), a key enzyme involved in lactate metabolism, is released in large amounts into the bloodstream when cells are damaged [17]. When AP causes tissue damage, the level of LDH in the blood increases, and it may also affect the normal metabolism and clearance of lactate, with a close relationship between the two [18]. Studies have shown that lactate and LDH have certain values in the condition assessment of some diseases, but there are relatively few studies on their dynamic changes in the early prediction of PRF in AP patients [19, 20].

This study aims to analyze a large sample of AP patients' dynamic changes in lactate and LDH within 24 h to explore their early predictive value for PRF, providing more valuable reference for clinical physicians in the early diagnosis and treatment decision-making of AP patients.

## Materials and methods

### Study design and data source

This retrospective observational study utilized data collected from patients with acute pancreatitis admitted to the Department of Gastroenterology at the First Affiliated Hospital of Nanchang University between January 2017 and December 2020. The information was sourced from a database of our center that is maintained prospectively. The hospital's ethics committee approved this study (No. 2011001). Given the retrospective nature of the study, informed consent from patients was waived.

### Patient selection

Data were collected from patients admitted to our hospital. Inclusion criteria were as follows: patients had to meet the diagnostic criteria for acute pancreatitis based on the 2012 Atlanta Classification, which required the presence of abdominal pain consistent with acute pancreatitis, serum amylase and/or lipase activity at least three times the upper limit of normal, and abdominal imaging showing pancreatic inflammation [3]. Additionally, patients were required to have complete records of at least one or multiple arterial blood lactate and serum LDH tests within 24 h of hospital admission. Exclusion criteria were as follows: patients with a pre-existing diagnosis of chronic renal failure or other known kidney diseases that could lead to irreversible kidney damage; patients with severe underlying diseases that could affect lactate and LDH metabolism, such as hereditary metabolic diseases, severe hematological disorders, or advanced-stage malignancies; and patients with incomplete clinical data, including basic information, biochemical indicators, treatment processes, or outcome data that would affect the analysis of relevant variables in this study.

### Data collection

For each patient, baseline demographic data including age, gender, etiology (categorized as biliary, alcoholic, hypertriglyceridemia, or other causes), and body mass index (BMI) were collected. Additionally, the Acute Physiology and Chronic Health Evaluation II (APACHE II) score upon patient admission, which is used to assess the severity of the patient's condition, was recorded. Blood lactate and LDH levels were collected at different time points within 24 h of hospitalization. Lactate was measured using a blood gas analyzer, and other biochemical indicators were completed in the hospital's laboratory under standardized procedures. The primary outcome measure was persistent renal failure, which was diagnosed if patients exhibited sustained increases in serum creatinine levels (≥ 1.9 mg/dl) for more than 48 h during hospitalization [3, 4].

### Grouping method

To reflect the dynamic changes in lactate and LDH on disease progression, the following grouping methods were applied: Patients were categorized into a lactate ≥ 2 mmol/L group if any lactate measurement within 24 h exceeded 2 mmol/L. Patients were categorized into an LDH ≥ 700 U/L group if any LDH measurement within 24 h exceeded 700 U/L. Based on the above grouping, further stratification was done to identify patients with both LDH ≥ 700 U/L and lactate ≥ 2 mmol/L, patients with either LDH ≥ 700 U/L or lactate ≥ 2 mmol/L, and patients with LDH less than 700 U/L and lactate less than 2 mmol/L [18].

### Statistical analysis

IBM SPSS Statistics software version 25.0 (Chicago, USA) was employed for data organization, preprocessing, and statistical analyses. Continuous variables were tested for normality, with normally distributed variables presented as mean ± standard deviation (x ± s) and non-normally distributed variables as median (interquartile range). Categorical variables were expressed as frequencies and percentages. For group comparisons, t-tests (for normally distributed and equal variance) or Mann–Whitney U tests (for non-normally distributed or unequal variance) were used for continuous variables, while chi-square tests or Fisher's exact probability method (when expected frequencies were less than 5) were applied for categorical variables. Logistic regression models were performed to predict risk factors with categorical dependent variables. Receiver Operating Characteristic (ROC) curves were plotted, and the area under the curve (AUC) was calculated to evaluate the predictive ability and accuracy of the models. Predictive metrics including sensitivity, specificity, positive predictive value, negative predictive value, positive likelihood ratio, and negative likelihood ratio were calculated based on the grouping of lactate and LDH levels to assess the risk of persistent renal failure. All statistical analyses were two-tailed, and *P* < 0.05 was regarded as statistically significant.

## Results

### Patient demographics and clinical characteristics

A total of 798 patients with acute pancreatitis were included in the study, divided into persistent renal failure (PRF) group (*N* = 111) and non-PRF group (*N* = 687). As shown in Table 1, the PRF group had a higher proportion of males (74% vs 63%, *P* = 0.031), with an average age of 47 years (IQR: 41–63) compared to 49 years (IQR: 36–61) in the non-PRF group (*P* = 0.041). The average BMI was 25 (IQR: 22–29) in the PRF group and 24 (IQR: 22–27) in the non-PRF group (*P* = 0.010). In terms of medical history, the PRF group had higher proportions of diabetes (22% vs 12%, *P* = 0.008), smoking (40% vs 31%, *P* = 0.066), and alcohol consumption (47% vs 33%, *P* = 0.003). Biochemical indicators showed significantly higher levels of White blood cell (WBC), Hematocrit (HCT), Alanine aminotransferase (ALT), Aspartate aminotransferase (AST), Lactate, LDH, Triglyceride (TG), Blood urea nitrogen (BUN), Creatinine, D-dimer, Median Systemic inflammatory response syndrome (SIRS), Median APACHE II, Median hospital days, Median ICU days, and Mortality in the PRF group.

**Table 1:** Demographic and clinical characteristics of patients with PRF versus non-PRF of acute pancreatitis (*n* = 798)

Variables	PRF	Non-PRF	*P*
	***N* = 111**	***N* = 687**	
Male, N (%)	82 (74)	435(63)	0.031
Median age, (IQR)	47 (41–63)	49 (36–61)	0.041
Median BMI, (IQR)	25 (22–29)	24 (22–27)	0.010
History of diabetes	24 (22)	85 (12)	0.008
Smoking	44 (40)	212(31)	0.066
Drinking alcohol	52 (47)	223 (33)	0.003
Etiology, N (%)			0.309
Biliary	42 (38)	305 (44)	0.196
Alcoholism	8 (7)	52 (8)	0.893
Hypertriglyceridemia	56 (51)	288 (42)	0.092
Idiopathic	5 (5)	33 (5)	0.891
WBC, × 103/μL	13.0 (9.4, 16.6)	12.3 (10.0, 18.3)	0.341
HCT, %	42.4 (37.9, 46.5)	45.6 (39.0, 51.5)	< 0.001
ALT, U/L	32.0 (18.0, 86.2)	36.0 (20.0, 95.0)	0.32
AST, U/L	37.0 (23.4, 72.2)	78.0 (47.0, 124.5)	< 0.001
Lactate, mmol/L	1.7 (1.2, 2.7)	3.2 (2.0, 5.0)	< 0.001
LDH, U/L	415.0 (290.5, 564.0)	883.0 (588.0, 1272.5)	< 0.001
TG, mmol/L	2.0 (1.0, 9.4)	3.4 (1.7, 13.7)	< 0.001
ALB, g/L	38.4 (34.0, 42.2)	33.7 (30.1, 38.5)	< 0.001
CK, U/L	88.0 (55.0, 165.0)	330.0 (147.5, 841.0)	< 0.001
GLU, mmol/L	8.7 (6.5, 12.6)	12.7 (8.2, 18.6)	< 0.001
BUN, mmol/L	5.2 (3.7, 7.3)	11.9 (8.9, 15.9)	< 0.001
Creatinine, mmol/L	67.1 (53.9, 86.6)	217.0 (149.6, 328.6)	< 0.001
D-dimer, mg/L	1.9 (0.9, 4.3)	4.7 (2.4, 7.9)	< 0.001
Median SIRS, (IQR)	3(2–3)	2 (1–3)	< 0.001
Median APACHEII, (IQR)	14 (11–17)	10 (7–12)	< 0.001
Median hospital days (IQR)	23(12–56)	10 (7–17)	< 0.001
Median ICU days, (IQR)	15 (8–34)	1 (0–7)	< 0.001
Mortality, N (%)	62 (56)	28 (4)	< 0.001

*AP* Acute pancreatitis, *N* Number, *BMI* Body Mass Index, *WBC* White blood cell, *HCT* Hematocrit, *ALT* Alanine aminotransferase, *AST* Aspartate aminotransferase, *Lactate* Lactic acid, *LDH* Lactate dehydrogenase, *TG* Triglyceride, *ALB* Albumin, *CK—MB* Creatine kinase – MB, *GLU* Glucose, *BUN* Blood urea nitrogen, *SIRS* Systemic inflammatory response syndrome, *APACHEII* Acute Physiology and Chronic Health Evaluation II, *ICU* Intensive Care Unit, *IQR* Inter Quartile Range

### Multivariate risk analysis

**Fig. 1:**
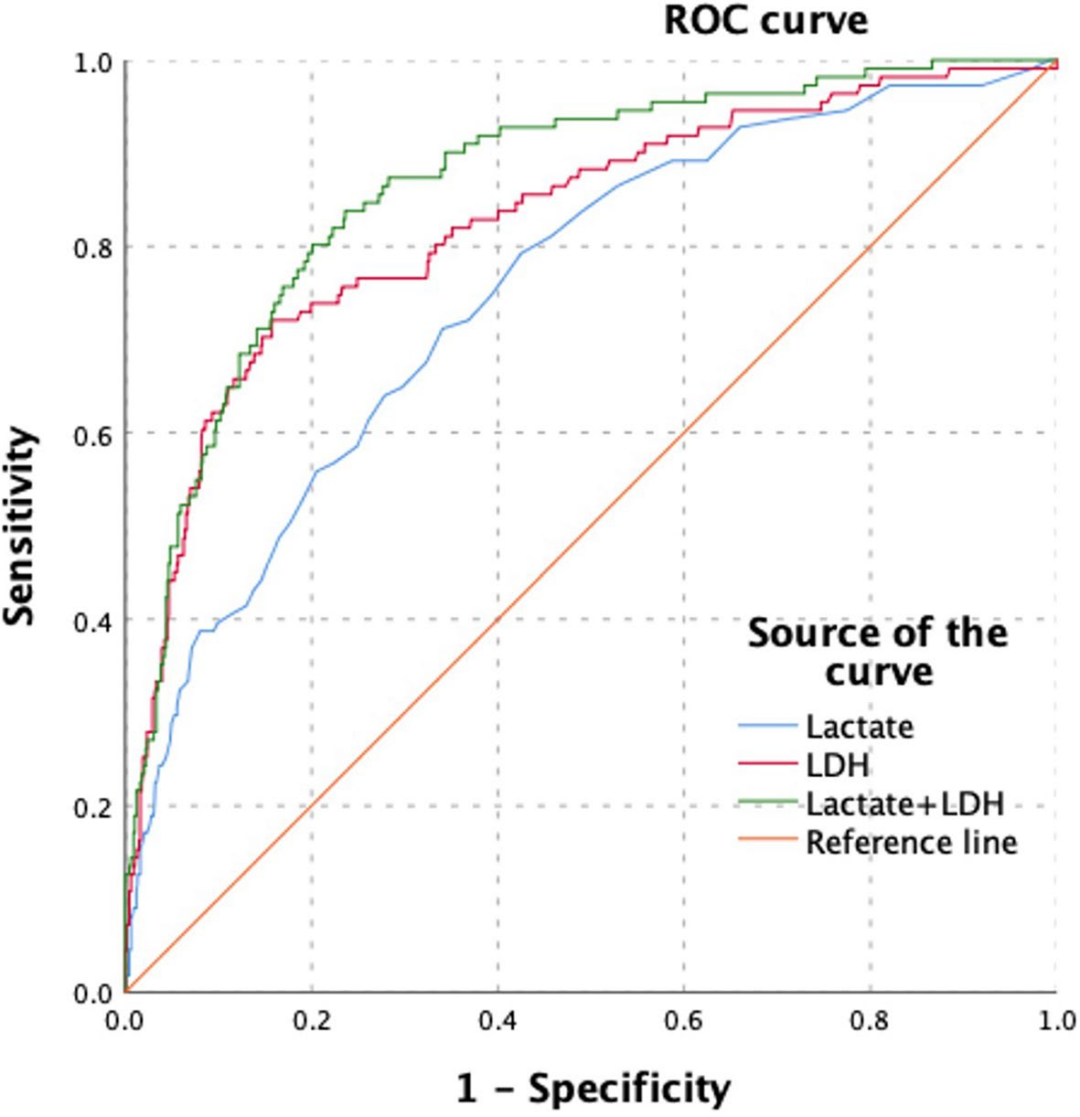
The figure shows the Receiver Operating Characteristic (ROC) curves for different biomarkers. The curves are plotted with Sensitivity on the y-axis and 1-Specificity on the x-axis. The blue curve represents the ROC curve for Lactate, the red curve for LDH, and the green curve for Lactate + LDH. The orange diagonal line is the Reference line. The curves illustrate the diagnostic performance of these biomarkers, with areas under the curves indicating their discriminatory power

Table 2 presents the results of the multivariate logistic regression analysis assessing the risk factors for PRF. Gender (β = −0.752, *P* = 0.084, OR = 0.471, 95% CI: 0.201–1.108), SIRS (β = 0.467, *P* = 0.027, OR = 1.594, 95% CI: 1.055–2.411), and APACHE II (β = 0.090, *P* = 0.035, OR = 1.094, 95% CI: 1.006–1.190) showed varying degrees of association. Lactate (β = 0.340, *P* < 0.001, OR = 1.405, 95% CI: 1.172–1.684) and LDH (β = 0.002, *P* < 0.001, OR = 1.002, 95% CI: 1.001–1.003) were the most significant risk factors. The AUC values for lactate, LDH, and the combined lactate + LDH were 0.752 (95% CI: 0.703–0.801), 0.828 (95% CI: 0.782–0.873), and 0.866 (95% CI: 0.830–0.902), respectively, with the highest diagnostic accuracy for the combined lactate + LDH indicator (Fig. 1 and Table 3).

**Table 2:** Multivariate analysis of risk factors for patients with PRF versus non-PRF of acute pancreatitis (*n* = 798)

Variable	β	SE	Wald	df	*P*—value	OR	95% CI of OR
SEX	−0.752	0.436	2.978	1	0.084	0.471	(0.201, 1.108)
Age	0.020	0.013	2.472	1	0.116	1.021	(0.995, 1.047)
BMI	0.033	0.042	0.627	1	0.429	1.034	(0.952, 1.123)
LAC	0.340	0.092	13.511	1	< 0.001	1.405	(1.172, 1.684)
LDH	0.002	0.000	21.863	1	< 0.001	1.002	(1.001, 1.003)
Diabetes History	−0.305	0.411	0.549	1	0.459	0.736	(0.325, 1.666)
Smoking History	−0.522	0.399	1.708	1	0.191	0.593	(0.277, 1.274)
Drinking History	−0.110	0.389	0.081	1	0.777	0.896	(0.418, 1.919)
HCT	−0.035	0.024	2.090	1	0.148	0.966	(0.921, 1.013)
AST	−0.001	0.001	0.277	1	0.598	0.999	(0.997, 1.001)
ALB	−0.045	0.032	2.024	1	0.155	0.956	(0.898, 1.017)
CK	0.000	0.000	0.431	1	0.511	1.000	(1.000, 1.001)
TG	0.017	0.018	0.869	1	0.351	1.017	(0.981, 1.054)
GLU	0.017	0.027	0.407	1	0.524	1.017	(0.965, 1.072)
D—dimer	−0.012	0.022	0.297	1	0.586	0.988	(0.946, 1.033)
SIRS	0.467	0.211	4.892	1	0.027	1.594	(1.055, 2.411)
APACHEII	0.090	0.043	4.451	1	0.035	1.094	(1.006, 1.190)

*BMI* Body Mass Index, *WBC* White blood cell, *HCT* Hematocrit, *ALT* Alanine aminotransferase, *AST* Aspartate aminotransferase, *Lactate* Lactic acid, *LDH* Lactate dehydrogenase, *TG* Triglyceride, *ALB* Albumin, *CK—MB* Creatine kinase – MB, *GLU* Glucose, *BUN* Blood urea nitrogen, *SIRS* Systemic inflammatory response syndrome, *APACHEII* Acute Physiology and Chronic Health Evaluation II, *β* Regression coefficient, *SE* Standard error, *Wald* Wald statistic, *df* Degrees of freedom, *P—value* Probability – value, *OR* Odds ratio, *OR* Odds ratio

**Table 3:** Prediction performance of lactate and LDH

Variables	AUC	95%CI	Threshold	Sensitivity	Specificity	Youden
Lactate	75.24%	(70.34% ~ 80.13%)	2.25	71.17%	65.94%	1.37
LDH	82.75%	(78.24% ~ 87.26%)	677.5	72.07%	84.28%	1.56
Lactate + LDH	86.63%	(83.01% ~ 90.24%)	-	84.21%	76.36%	1.60

*AUC* Area under curve, *CI* Confidence interval, *LDH* Lactate dehydrogenase

### Stratified analysis based on LDH and lactate levels

**Fig. 2:**
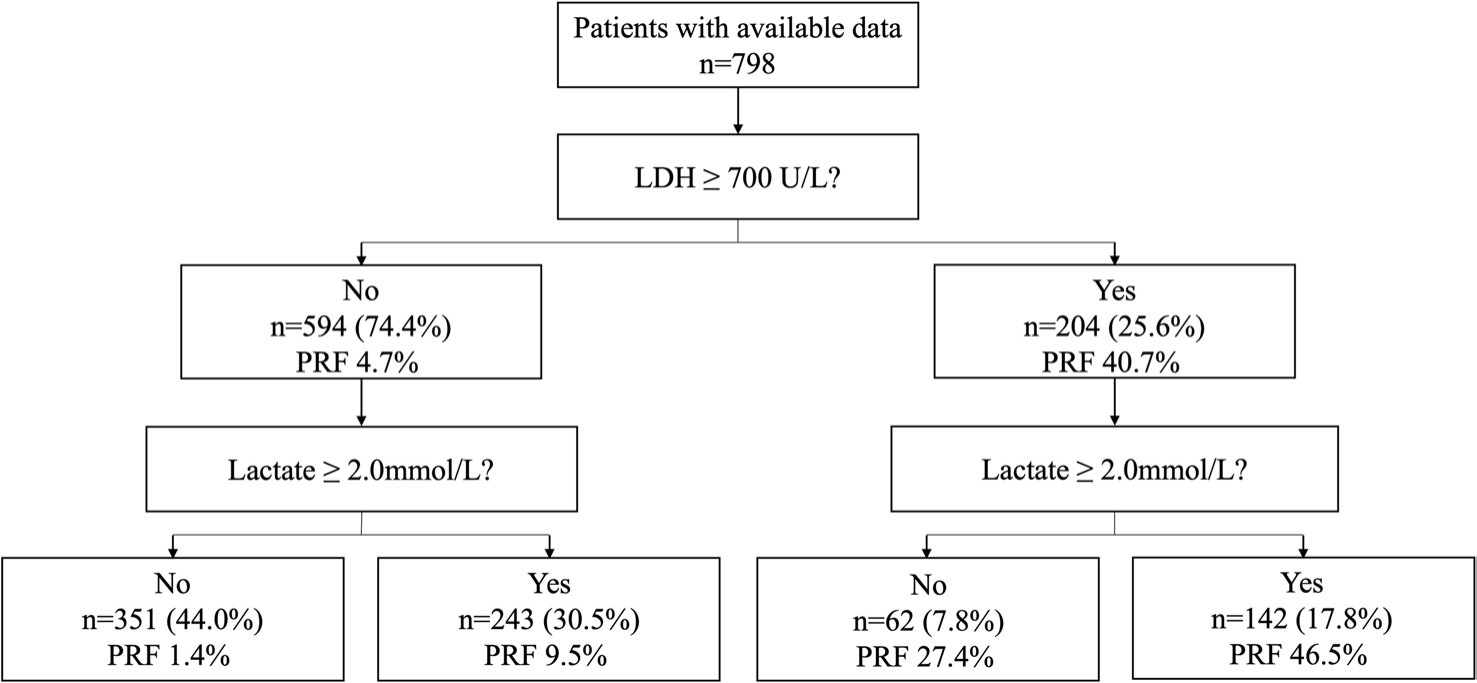
The figure is a flow chart depicting the distribution of patients with available data (*n* = 798) based on their Lactate Dehydrogenase (LDH) and Lactate levels

Stratified analysis based on LDH and lactate levels revealed significant differences in the incidence of PRF across different groups. Patients with LDH ≥ 700 U/L had a PRF incidence of 40.7% (*n* = 204), while those with LDH < 700 U/L had an incidence of 4.7% (*n* = 594). Further stratification within the LDH < 700 U/L group showed that patients with lactate ≥ 2.0 mmol/L had a PRF incidence of 9.5% (*n* = 243), while those with lactate < 2.0 mmol/L had an incidence of 1.4% (*n* = 351). Within the LDH ≥ 700 U/L group, patients with lactate ≥ 2.0 mmol/L had a PRF incidence of 46.5% (*n* = 142), while those with lactate < 2.0 mmol/L had an incidence of 27.4% (*n* = 62) (Fig. 2).

### Characteristics of different risk groups

The high-risk group (LDH > 700 U/L and lactate > 2 mmol/L) had a PRF incidence rate of 46.5% (66/142), the medium-risk group (LDH > 700 U/L or lactate > 2 mmol/L) had a PRF incidence rate of 13.1% (40/305), and the low-risk group (LDH < 700 U/L and lactate < 2 mmol/L) had a PRF incidence rate of only 1.4% (5/351) (Table 4). Predictive indicators calculated showed that with LDH > 700 U/L or lactate > 2 mmol/L as the positive criterion, the sensitivity for predicting PRF was 95.5%, specificity was 79.4%, positive predictive value was 13.1%, negative predictive value was 99.3%, positive likelihood ratio was 4.64, and negative likelihood ratio was 0.057. With LDH > 700 U/L and lactate > 2 mmol/L as the positive criterion, the sensitivity was 59.5%, specificity was 92.3%, positive predictive value was 46.5%, negative predictive value was 97.7%, positive likelihood ratio was 7.73, and negative likelihood ratio was 0.44 (Table 5).

**Table 4:** Clinical characteristics and disease—related indicators of different risk groups in acute pancreatitis

Variables	Low-risk group (LDH < 700 U/L and lactate < 2 mmol/L)	Medium-risk group (LDH > 700 U/L or lactate ≥ 2 mmol/L)	High-risk group (LDH > 700 U/L and lactate ≥ 2 mmol/L)	*P*
	***N* = 351**	***N* = 305**	***N* = 142**	
Male, N (%)	231 (66)	186 (61)	100 (70)	0.131
Median age, (IQR)	49 (36–62)	50 (38–64)	46 (39–59)	0.128
Etiology, N (%)				
Biliary	156 (44)	142 (47)	49 (35)	0.051
Alcoholism	26 (7)	20 (6)	14 (10)	0.465
Hypertriglyceridemia	140 (40)	129 (42)	75 (53)	0.030
Idiopathic	22 (6)	12 (4)	4 (3)	0.183
Median SIRS, (IQR)	2 (1–2)	2 (2–3)	3 (2–3)	< 0.001
Median APACHEII, (IQR)	8 (6–11)	10 (7–13)	13 (10–16)	< 0.001
MAP, N (%)	121 (34)	45 (15)	4 (3)	< 0.001
MSAP, N (%)	140 (40)	98 (32)	15 (11)	< 0.001
SAP, N (%)	90 (26)	162 (53)	123 (87)	< 0.001
Median hospital days (IQR)	9 (6–13)	11 (7–20)	23 (13–51)	< 0.001
Median ICU days, (IQR)	0 (0–3)	2 (0–9)	13 (5–31)	< 0.001
APFC, N (%)	122 (35)	103 (34)	20 (14)	< 0.001
ANC, N (%)	66 (19)	118 (39)	110 (78)	< 0.001
Renal failure, N (%)	17 (5)	68 (22)	83 (59)	< 0.001
Persistent renal failure, N (%)	5 (1)	40 (13)	66 (47)	< 0.001
Respiratory failure, N (%)	146 (42)	205 (67)	131 (92)	< 0.001
Persistent respiratory failure, N (%)	87 (25)	153 (50)	117 (82)	< 0.001
PN, N (%)	92 (26)	127 (42)	110 (78)	< 0.001
IPN, N (%)	15 (4)	46 (15)	65 (46)	< 0.001
OF, N (%)	150 (43)	207 (68)	131 (92)	< 0.001
POF, N (%)	90 (26)	162 (53)	123 (87)	< 0.001
Persistent MOF, N (%)	4 (1)	36 (12)	61 (43)	< 0.001
Mortality, N (%)	4 (1)	35 (12)	51 (36)	< 0.001

*LDH* Lactate dehydrogenase, *SIRS* Systemic inflammatory response syndrome, *APACHEII* Acute Physiology and Chronic Health Evaluation II, *MAP* Mean arterial pressure, *MSAP* Moderately severe acute pancreatitis, *SAP* Severe acute pancreatitis, *APFC* Acute peripancreatic fluid collection, *ANC* Abnormal pancreatic parenchymal collection, *PN* Pancreatic necrosis, *IPN* Infected pancreatic necrosis, *OF* Organ failure, *POF* Persistent organ failure, *MOF* Multiple organ failure

**Table 5:** Risk stratification and predictive performance of lactate and LDH

Risk Category	Low-risk group	Medium-risk group	High-risk group
Sensitivity	Reference	95.5%	59.5%
Specificity	Reference	79.4%	92.3%
Positive Predictive Value	Reference	13.1%	46.5%
Negative Predictive Value	Reference	99.3%	97.7%
Positive Likelihood Ratio	Reference	4.64	7.73
Negative Likelihood Ratio	Reference	0.057	0.44

Based on the results, patients were categorized into low-risk (LDH < 700 U/L and lactate < 2 mmol/L), medium-risk (LDH > 700 U/L or lactate ≥ 2 mmol/L), and high-risk (LDH > 700 U/L and lactate ≥ 2 mmol/L) groups (Table 4). The high-risk group had a significantly higher incidence of PRF compared to the low-risk group (OR = 7.752, 95% CI: 4.839–12.418).

## Discussion

This study aimed to investigate the predictive value of the changes of lactate and its metabolic indicator (LDH) within 24 h in patients with acute pancreatitis for persistent renal failure. The results showed that different combinations of lactate and LDH levels had varying degrees of predictive ability for persistent renal failure. When using LDH ≥ 700 U/L or lactate ≥ 2 mmol/L as the standard, it had a relatively high sensitivity (95.37%), suggesting that this standard could effectively identify most patients who may develop persistent renal failure. This is of great significance for the early detection of high-risk patients in clinical practice. In clinical work, it can be used as a warning signal, prompting doctors to conduct closer monitoring of renal function and take active preventive measures for patients. When using LDH ≥ 700 U/L and lactate ≥ 2 mmol/L as the standard, the specificity was relatively high (92.26%), which means that this standard had a high accuracy in excluding patients without persistent renal failure, helping to reduce unnecessary medical interventions and waste of medical resources.

The positive and negative predictive values under different standards also provided strong evidence for clinical decision-making. For example, when LDH ≥ 700 U/L and lactate ≥ 2 mmol/L, the positive predictive value (44.68%) indicated that when patients met these two conditions simultaneously, the possibility of developing persistent renal failure was relatively high, and doctors should attach great importance to it and actively adjust the treatment plan. The negative predictive value (97.70%) indicated that when the levels of lactate and LDH in patients did not reach this standard simultaneously, persistent renal failure could be basically excluded with confidence. The positive and negative likelihood ratios further quantified the value of these indicators in diagnosis from different perspectives, helping clinicians better understand the significance of test results for disease diagnosis [21–23].

In the pathophysiological process of acute pancreatitis, the inflammatory response-induced microcirculation disturbance and tissue hypoxia are the key links. The local inflammation of the pancreas leads to increased vascular permeability and changes in blood rheology, resulting in insufficient pancreatic tissue perfusion and cells in a hypoxic state [24]. In this case, cell metabolism shifts from aerobic to anaerobic metabolism, and lactate is produced in large quantities as a product of anaerobic metabolism [25]. The dynamic change of lactate level within 24 h can reflect this change in metabolic state, and its continuous increase means that tissue hypoxia persists or worsens [26].

LDH, as an enzyme widely existing in cells, is released into the blood when cells are damaged. In acute pancreatitis, the inflammatory damage of pancreatic tissue causes many cells to be destroyed, and the LDH level increases. At the same time, the increased LDH may be involved in the abnormal process of lactate metabolism, further affecting the metabolism and clearance of lactate. When the dynamic changes of lactate and LDH occur abnormally at the same time, it indicates that the metabolic disorder and tissue damage of the body have reached a certain extent. The kidneys, being sensitive organs to changes in blood perfusion and metabolism, are more likely to be affected, thereby increasing the risk of persistent renal failure [7, 16, 19].

Previous studies mostly focused on the assessment of the prognosis of acute pancreatitis by traditional scoring systems (such as APACHE II score) or single biomarkers (such as CRP), but these methods have certain limitations in predicting persistent renal failure. Compared with these studies, this study focused on the early changes of lactate and LDH, providing more valuable information for the prediction of persistent renal failure in patients with acute pancreatitis from a new perspective. Although some previous studies involved the detection of lactate or LDH, most of them were single-time-point measurements and failed to fully consider the impact of their dynamic changes within 24 h on the disease progression. This study more accurately captured the risk change of patients developing persistent renal failure during the disease progression by dynamically observing the changes of lactate and LDH, providing a timelier basis for early clinical prediction [12, 13, 27].

Despite the meaningful results obtained in this study, there are still some limitations. First, this is a retrospective study, and information bias is inevitable. The requirement for at least one lactate or LDH measurement within 24 h of admission may have systematically excluded patients with very mild disease (deemed by physicians not to warrant frequent testing) or those with extremely severe disease (who died early or were transferred to the ICU before multiple tests could be performed), potentially limiting the generalizability of our findings. Future prospective studies with prespecified uniform time points for testing (e.g., admission, 6 h, 12 h, 24 h) are recommended to standardize data collection and reduce such bias. Second, the definition of PRF was based on an absolute creatinine threshold (≥ 1.9 mg/dl for > 48 h), which, while consistent with some AP-specific studies, differs from the current international KDIGO guidelines that emphasize relative changes in creatinine (e.g., an increase of ≥ 0.3 mg/dl within 48 h or 1.5 times the baseline within 7 days) and urine output. The use of an absolute threshold may not accurately identify patients with low baseline creatinine who experience significant relative changes. Future studies should consider adopting the KDIGO criteria to evaluate whether this affects the predictive performance of lactate and LDH. Third, our grouping was based on "any time" exceedance of thresholds within 24 h. We acknowledge that peak levels, trends (e.g., sustained increase, fluctuation, decrease), or time-weighted averages, such as lactate clearance, may contain more prognostic information than a single exceedance. The failure to analyze these dynamically changing patterns is a limitation of the current study. Fourth, in the multivariate model, the OR for LDH as a continuous variable was 1.002 (95% CI: 1.001–1.003), which, although statistically significant, is very close to 1, indicating a very small increase in risk per 1 U/L increase. The clinical significance of LDH as a continuous variable should be interpreted with caution. The categorical approach (e.g., ≥ 700 U/L) used in stratified analysis is likely more intuitive and practical for clinical decision-making. Fifth, although we attempted to adjust for some confounding factors, other important potential contributors to AKI/PRF in AP, such as blood pressure levels, occurrence of shock, use of nephrotoxic drugs (e.g., certain antimicrobials, NSAIDs), or exposure to contrast media, were not included in the analysis due to limitations in retrospective data availability. Additionally, urine output data was not compared. These factors should be considered in future studies. Sixth, we excluded patients with pre-existing chronic kidney disease (CKD). As CKD is a well-established risk factor for AKI, the conclusions of our study may not be generalizable to patients with underlying kidney disease. Finally, this study only focused on lactate and LDH. The pathogenesis of AP is complex, and other biomarker combinations or indicators (e.g., inflammatory factors, microcirculation-related markers) might improve predictive accuracy. Future research should explore multi-indicator combined prediction models [4, 5, 9].

Furthermore, while we proposed a risk stratification (low, medium, high), specific recommendations for differentiated management based on these strata require validation through interventional studies. For high-risk patients (LDH > 700 U/L and lactate ≥ 2 mmol/L), enhanced monitoring of renal function (e.g., frequent serum creatinine and urine output assessment), aggressive but careful fluid resuscitation to optimize perfusion while avoiding fluid overload, avoidance of nephrotoxic medications, and early nephrology consultation might be considered, although evidence supporting that these specific measures improve outcomes in this context is needed. Future research is recommended to explore and validate intervention strategies based on this risk stratification to determine if it can effectively reduce the incidence of PRF in high-risk AP patients and truly improve prognosis.

## Conclusion

In conclusion, this study indicates that the dynamic changes of lactate and LDH within 24 h have a certain predictive value for persistent renal failure in patients with acute pancreatitis. The categorical thresholds (LDH ≥ 700 U/L and/or lactate ≥ 2 mmol/L) provide a practical tool for risk stratification. These findings need to be further validated and refined in future prospective studies, particularly those incorporating KDIGO criteria, dynamic trend analysis, and additional clinical risk factors, to provide more accurate prediction tools and optimized treatment strategies for clinical practice.

## Data Availability

All data generated or analyzed during this study are included in this published article [and its supplementary information files].

## Abbreviations

AP: Acute pancreatitis
PRF: Persistent renal failure
LDH: Lactate dehydrogenase
SIRS: Systemic inflammatory response syndrome
APACHE II: Acute physiology and chronic health evaluation II
BISAP: Bedside index for severity in acute pancreatitis
CRP: C-reactive protein
PCT: Procalcitonin
AKI: Acute kidney injury
WBC: White blood cell
HCT: Hematocrit
ALT: Alanine aminotransferase
AST: Aspartate aminotransferase
TG: Triglyceride
BUN: Blood urea nitrogen
ICU: Intensive care unit
IQR: Interquartile range
ROC: Receiver operating characteristic
AUC: Area under the curve
OR: Odds ratio
CI: Confidence interval
