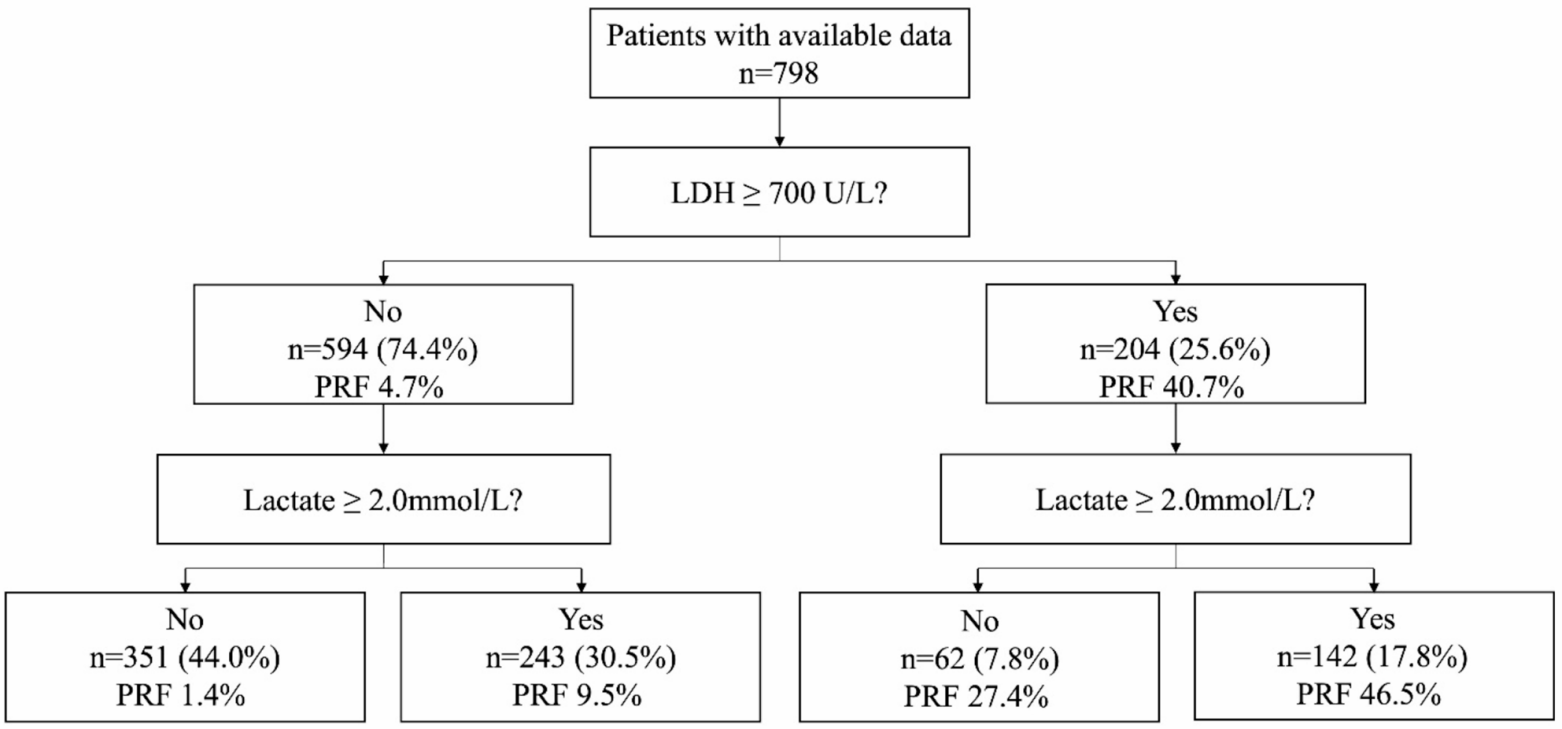# Correction: Early lactate and its metabolism for predicting persistent renal failure in patients with acute pancreatitis: a retrospective observational study

**DOI:** 10.1186/s12876-025-04514-6

**Published:** 2025-12-08

**Authors:** Jianhua Wan, Huajing Ke, Wenhua He, Yin Zhu, Nonghua Lu, Liang Xia

**Affiliations:** Department of Gastroenterology, Jiangxi Provincial Key Laboratory of Digestive Diseases, Jiangxi Clinical Research Center for Gastroenterology, Digestive Disease Hospital, The First Affiliated Hospital, Jiangxi Medical College, Nanchang University, 17 Yongwaizheng Street, Nanchang, Jiangxi 330006 People’s Republic of China

**Correction: BMC Gastroenterol 25, 811 (2025)**

**https://doi.org/10.1186/s12876-025-04413-w**

Following publication of the original article it was reported that the incorrect Fig. 2 was published. However, the caption was correct.

The incorrect and correct versions of Fig. 2 are given below. The original article has been corrected.

**Incorrect Fig. 2**.


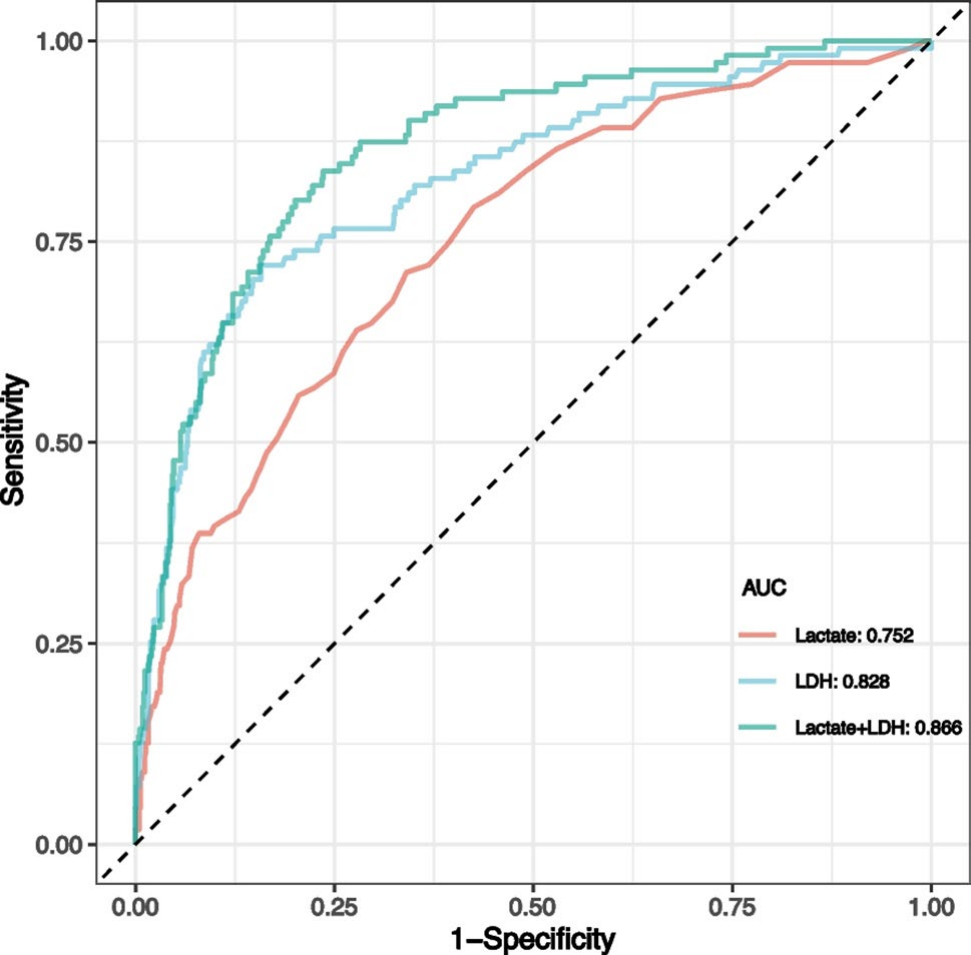


**Correct Fig. 2**.